# Treatment planning comparison of IMPT, VMAT and 4π radiotherapy for prostate cases

**DOI:** 10.1186/s13014-016-0761-0

**Published:** 2017-01-11

**Authors:** Angelia Tran, Jingjing Zhang, Kaley Woods, Victoria Yu, Dan Nguyen, Gary Gustafson, Lane Rosen, Ke Sheng

**Affiliations:** 1 Department of Radiation Oncology, University of California, Los Angeles, 200 Medical Plaza Way, Suite B265, Los Angeles, CA 90095 USA; 2 Department of Radiation Oncology, Beaumont Health System, Royal Oak, USA; 3 Department of Radiation Oncology, Willis-Knighton Cancer Center, Shreveport, USA

**Keywords:** Prostate cancer, 4π radiotherapy, Intensity modulated proton therapy, Volumetric modulated arc therapy

## Abstract

**Background:**

Intensity-modulated proton therapy (IMPT), non-coplanar 4π intensity-modulated radiation therapy (IMRT), and volumetric-modulated arc therapy (VMAT) represent the most advanced treatment methods based on heavy ion and X-rays, respectively. Here we compare their performance for prostate cancer treatment.

**Methods:**

Ten prostate patients were planned using IMPT with robustness optimization, VMAT, and 4π to an initial dose of 54 Gy to a clinical target volume (CTV) that encompassed the prostate and seminal vesicles, then a boost prescription dose of 25.2 Gy to the prostate for a total dose of 79.2 Gy. The IMPT plans utilized two coplanar, oblique scanning beams 10° posterior of the lateral beam positions. Range uncertainties were taken into consideration in the IMPT plans. VMAT plans used two full, coplanar arcs to ensure sufficient PTV coverage. 4π plans were created by inversely selecting and optimizing 30 beams from 1162 candidate non-coplanar beams using a greedy column generation algorithm. CTV doses, bladder and rectum dose volumes (V40, V45, V60, V65, V70, V75, and V80), R100, R50, R10, and CTV homogeneity index (D95/D5) were evaluated.

**Results:**

Compared to IMPT, 4π resulted in lower anterior rectal wall mean dose as well as lower rectum V40, V45, V60, V65, V70, and V75. Due to the opposing beam arrangement, IMPT resulted in significantly (*p* < 0.05) greater femoral head doses. However, IMPT plans had significantly lower bladder, rectum, and anterior rectal wall max dose. IMPT doses were also significantly more homogeneous than 4π and VMAT doses.

**Conclusion:**

Compared to the VMAT and 4π plans, IMPT treatment plans are superior in CTV homogeneity and maximum point organ-at-risk (OAR) doses with the exception of femur heads. IMPT is inferior in rectum and bladder volumes receiving intermediate to high doses, particularly to the 4π plans, but significantly reduced low dose spillage and integral dose, which are correlated to secondary cancer for patients with expected long survival. The dosimetric benefits of 4π plans over VMAT are consistent with the previous publication.

## Background

It is estimated that in the year 2015, there will be around 220,800 new cases of prostate cancer and around 27,540 deaths. Prostate cancer is the second most common cancer and the second leading cause of cancer death for men in the United States [1]. External beam radiation therapy is commonly used to treat prostate cancer. Studies have shown the benefits of 76 Gy or higher conventionally fractionated treatments, although there is a substantial risk of gastrointestinal toxicity, particularly stemming from the rectum dose [2, 3]. In these cases, radiation doses better conforming to the prostate are necessary to reduce possible rectal complications.

The use of charged particle beams, such as proton, demonstrates strong potential for highly conformal dose distribution. The Bragg peaks of proton beams allow extremely localized dose delivery at a precise depth with no exit dose after the distal tail and secondary particles. However, since most targets are larger than the Bragg peaks, a Spread-Out-Bragg-Peak (SOBP) must be created to homogeneously cover the target laterally and in the beam direction. A range-shifter wheel is typically used to modulate the incident proton energy for varying depths. The proton beams are further broadened by high-Z scatter foils and then collimated to the size of the target. To compensate for the surface contour of the patient, tissue composition and shape of the target, a custom compensator is made for each patient. With these additional devices, passive scattering delivers a number of individual Bragg peaks of different depths and weighted to achieve the SOBP. Although this technique has attracted worldwide interest, it is considered a simple method with considerable limitations [4] including low dose conformity, secondary particles including neutrons that increase patient integral dose and the logistic hurdles associated with devices needed for individual patients.

Active scanning is a development that can be automatically controlled, allowing proton beams to achieve a more efficient complete dose delivery [5]. To cover a target, each beam is scanned laterally across the target using magnetic fields in a technique called Pencil Beam Scanning (PBS) [6]. PBS enables state of the art intensity modulated proton therapy (IMPT), which is analogous to the intensity modulated radiation therapy (IMRT) that inversely optimize *all beams* to deliver a uniform dose to the target while individual beams only deliver a partial heterogeneous dose [7].

For photon therapy, Volumetric Modulated Arc Therapy (VMAT) is a widely adopted technique with advantages over conventional step-and-shoot Intensity-Modulated Radiation Therapy (IMRT), namely its delivery efficiency at equivalent dosimetry [8–10]. VMAT is unable to achieve the organ-at-risk (OAR) dose sparing demonstrated by proton therapy due to proton’s advantageous Bragg peaks [11–13]. However, photon therapy has the advantage of being a much more cost effective and widespread treatment modality. 4π radiotherapy is a non-coplanar IMRT technique that has demonstrated superior OAR dose sparing compared to VMAT in various tumor sites, including the prostate [14–18]. There is an increasing interest in comparing the non-coplanar 4π treatment to the state-of-the-art proton prostate therapy for relative dosimetric benefit. Here, we study the dosimetric performance of IMPT proton compared to photon VMAT and 4π therapy for prostate cancer.

## Methods

### Patients

This retrospective study was approved by the Internal Review Board of the Willis-Knighton Health System. Ten prostate patients were selected, each with an initial dose of 54 Gy to a clinical target volume (CTV) encompassing the prostate and seminal vesicles, then a boost of 25.2 Gy to a CTV encompassing only the prostate for a total dose of 79.2 Gy delivered in fractions of 1.8 Gy. The patients were then planned using the IMPT technique described as follows.

### IMPT planning

The treatment plans for the ten patients were generated with the IBA ProteusOne compact system beam model on the RayStation researh version 4.99.1 (RaySearch Laboratories, Stockholm, Sweden) with automatic spot spacing and spot placing. IMPT plans used two coplanar, oblique beams angled 10° posterior of the lateral beam positions. A pencil beam algorithm was used for proton dose calculation with 3 × 3 × 3 mm^3^ dose grid. The IBA ProteusOne compact gantry with C230 cyclotron has a 70–226 MeV energy range. The spot size in air is 3.5 mm at the max energy and 7.6 mm at the lowest energy. Spot size variation with gantry angle is less than 5%. A maximum of 0.5 cm uniform setup error and a range uncertainty of 3.5% were used in the robustness setting for optimization. Since the concept of PTV was not used in IMPT, dosimetric analysis of the target was focused on the clinical target volume (CTV). The dose objectives used for all treatment plans are shown in Table 1, with the CTV dose normalized at 100% of prescription dose delivered to 100% of the volume.

**Table 1:** Structure dose constraints used for IMPT, 4π, and VMAT planning

Structure	Objectives
PTV	100% of Rx to 100% of CTV
Bladder	V70 < 20%
	V40 < 60%; V45 < 50%; V60 < 40%; V70 < 20%; V75.6 <
Rectum	10%; V78–80 < 5%
Femoral heads	V45 < 50%; max dose < 50Gy
Sigmoid colon	max dose < 50Gy
Small bowel	max dose < 50Gy
Anterior rectal wall	V70 < 40%

### VMAT planning

The IMPT treatments were re-planned using VMAT (RapidArc, Eclipse Treatment Planning System version 13, Varian) with 2.5 × 2.5 × 2.5 mm^3^ dose grid. Both photon treatments used the PTVs for planning but then normalized to the CTV to be consistent with the IMPT plans. For the X-ray plans, these PTVs have a 5 mm posterior margin and 6 mm in all other directions. Each plan used two full coplanar arcs to ensure good PTV coverage. To match the proton plan target coverage, VMAT plans were normalized for 100% of the prescription dose covering 100% of the CTVs. With this primary prescription satisfied, on average, 97.3% of PTV is covered by 100% of the prescription dose. The collimator was rotated 90° between the arcs. Optimization objectives for VMAT planning were the same constraints used in IMPT planning (Table 1) or lower, if possible, for normal tissues. PTV hot spots were constrained to be as low as possible.

### 4π radiotherapy

4π radiotherapy was developed to incorporate non-coplanar beams distributed on the 4π spherical surface, thus the name, in IMRT optimization. 4π optimization begins with a candidate pool of 1162 non-coplanar beams, each 6° apart in the 4π solid angle space. Using a computer assisted design (CAD) model of the Varian TrueBeam machine and a 3D human surface model, each angle is simulated and subsequently eliminated if a collision is predicted between the gantry and the couch or patient [19]. The remaining beams were divided into 5 × 5 mm^2^ beamlets, whose dose was calculated using convolution/superposition and Monte Carlo calculated 6MV polyenergetic kernels as described previously [20, 21]. The dose calculation resolution is 2.5 × 2.5 × 2.5 mm^3^. Inverse optimization is performed by using a greedy column generation algorithm to iteratively select 30 non-coplanar beams with integrated fluence map optimization [22]. The 30 beam angles consisted of 24–30 couch kicks for the 10 patients. The beam angles were then imported into Eclipse to recalculate the IMRT dose, creating a clinically deliverable plan that can be directly compared to the IMPT and VMAT plans. The 4π optimization objectives used in Eclipse were identical to VMAT constraints as described above, including normalization for the CTV dose. On average, 99.3% of PTV is covered by 100% of the prescription dose.

### Dose comparison

Various dose metrics were evaluated for comparison of the IMPT, VMAT, and 4π plans. Table 1 lists the planning objectives used for all treatment planning methods. Metrics used for planning objectives were calculated and compared between planning techniques, including V40, V45, V60, V70, V75.6, V80 of the rectum, V70 of the bladder, mean, and max doses for organs at risk. Multivariate regression was performed on these OAR metrics to determine the influence of the OAR volume. Because the concept of PTV is no longer used in IMPT prostate planning, CTV coverage was compared using mean, max doses, and CTV homogeneity index, which was evaluated by calculating the D95 to D5 ratio. R50 and R10, which were defined as the 50 and 10% isodose volume to evaluation CTV ratios, were also calculated to examine high dose and low dose spillage, respectively. Since PTV was not used in the IMPT plans, the standard conformity index of the ratio between the 100% isodose volume and the PTV did not apply. Instead, to quantify the 100% isodose volume, we calculated R100, which is the ratio between 100% isodose volume and the CTV.

## Results

**Fig. 1:**
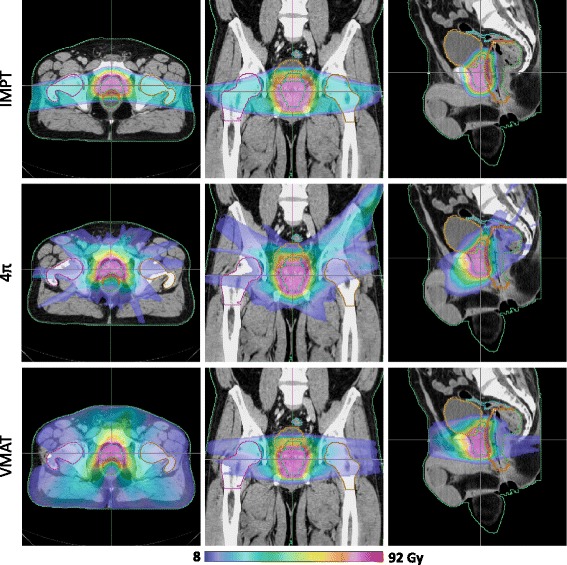
Isodose colorwash of a typical patient planned using IMPT, 4π, and VMAT plans

**Fig. 2:**
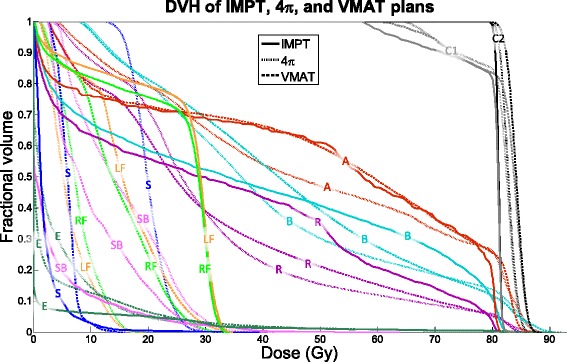
DVH of an example case for IMPT, 4π, and VMAT plans. A = anterior rectal wall, B = bladder, C1 = CTV 1, C2 = CTV 2, E = external, LF = left femoral head, RF = right femoral head, R = rectum, S = sigmoid, SB = small bowel

Isodose and dose volume histogram (DVH) comparisons between the three treatment modalities for a representative example case are shown in Figs. 1 and 2. As one would expect, the lateral beam angles used by the IMPT plans delivered substantially greater dose to the femoral heads than photon plans delivering beams from vastly more beam orientations. It is also worth to note the oblique dose distribution patterns resulted from 4π non-coplanar beams, in comparison to the coplanar VMAT and proton plans. Subsequently, the 4π femoral head doses are also significantly lower than those of VMAT. However, IMPT resulted in more homogeneous CTV coverage, reducing the hot spots visible in the 4π and VMAT dose in Fig. 1 as well as the CTV DVHs in Fig. 2.

**Fig. 3:**
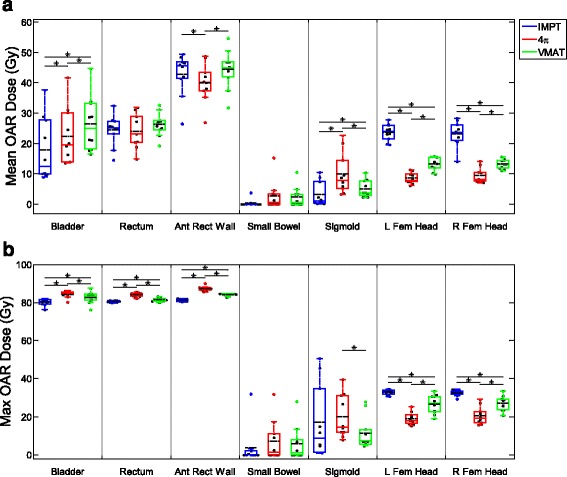
Box and dot plots of OAR mean and max doses. *p < 0.05

**Fig. 4:**
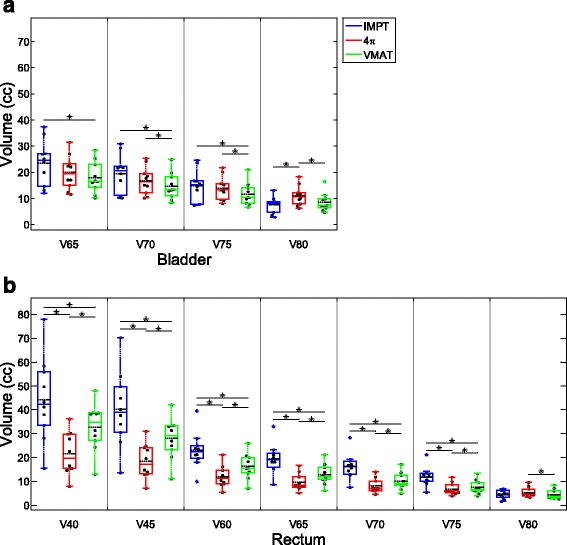
Box and dot plots of bladder and rectum dose volume metrics. *p < 0.05

**Fig. 5:**
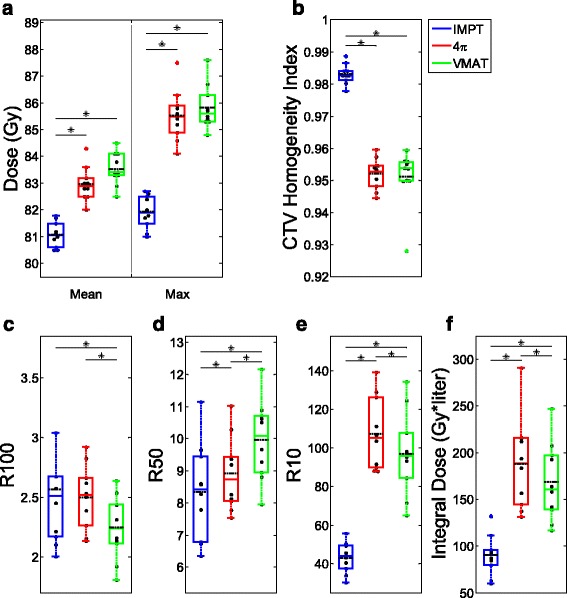
Box and dot plots of CTV metrics. *p < 0.05

The OAR and CTV dose metrics are shown in Figs. 3, 4, and 5 as boxplots for each patient overlaid with boxplots summarizing the data. The central colored line of the boxplots represents the median, with the edges representing the 25^th^ and 75^th^ percentiles. The whiskers show the range of data excluding outliers. The central, dashed black line represents the mean. Wilcoxon signed rank tests were performed between each pair of treatment modalities. Significant differences (*p* < 0.05) between treatment modalities are annotated in Figs. 3, 4, and 5 with asterisks.

Mean doses of the bladder and sigmoid colon and max doses for the bladder, rectum, and anterior rectal wall were lowest with IMPT planning. 4π had the lowest mean dose for the anterior rectal wall and the femoral heads and the lowest max dose for the femoral heads. VMAT did not outperform both 4π and IMPT in any OAR mean or max dose (Fig. 3). Of the specific dose metric constraints for the bladder, VMAT had the lowest V70 and V75. 4π achieved the lowest dose volumes for all rectum metrics except for V80 (Fig. 4). IMPT is superior in almost all of the CTV dose metrics, showing more homogeneous dose distribution in the CTV. IMPT had the least intermediate and low dose spillage (R50 and R10), as well as integral dose. However, the 100% isodose volume was lowest with VMAT plans.

**Fig. 6:**
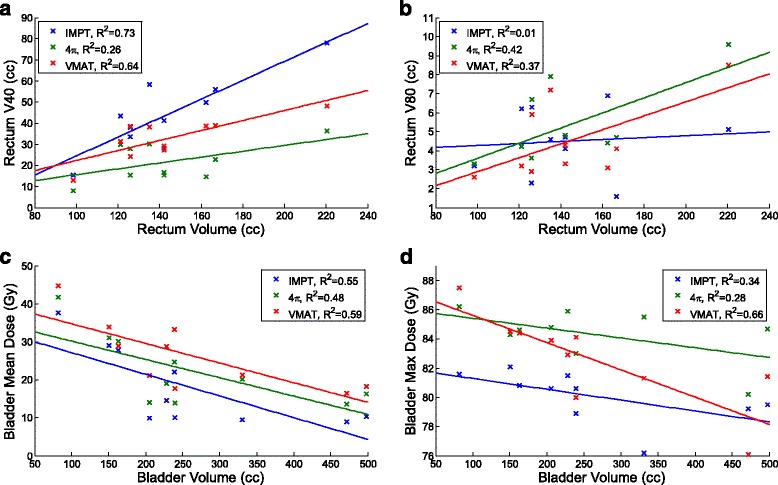
Multivariate regression analysis on rectum V40, rectum V80, bladder mean, and bladder max dose

Figure 6 shows the results of the multivariate regression analysis for dose metrics and OAR volumes. Rectum V40 increases with increasing rectum volume for all three techniques but it appears to increase more with IMPT than the X-ray counter parts. However, rectum V80 of IMPT increases slower than that of VMAT and 4π. As expected, the average bladder doses decrease for all three planning methods but the maximum doses also decrease with increasing bladder volume.

## Discussion

Proton therapy is attractive due to the unique physical properties of the heavy charged particles that deliver the majority of dose in sharp Bragg peaks and leave no exit dose. On the other hand, the side by side dosimetric comparison between proton therapy and the best of photon therapy has rarely been performed. In a dosimetric comparison between 3D conformal proton therapy (CPT) and IMRT, Trofimov et al. concluded that IMRT resulted in superior bladder sparing and similar rectum sparing compared to 3D CPT, which is superior in reducing the low dose spillage [23]. The same study also pointed out that the lack of dose conformity in 3D CPT would be overcome with the use of scanning pencil beam and intensity modulated proton therapy (IMPT). With the improvement of proton therapy techniques, PBS proton has gradually replaced passive scatter due to its superior dose shaping capability. In our comparison, state of the art PBS based IMPT was used.

At the same time, VMAT has evolved to be the mainstay therapy method for the prostate because of good dosimetry quality and superior delivery efficiency, compared to static beam IMRT. There has been a notion that VMAT may be the ultimate IMRT method for the prostate [24] and static beam IMRT will be completely phased out. 4π radiotherapy revived non-coplanar IMRT methods by providing a mathematical tool for combined beam orientation and fluence map optimization. This method was shown to be advantageous to coplanar VMAT for almost all disease sites including the prostate and yet is deliverable on existing C-arm linacs. In light of the technical improvement in both photon and proton techniques, revisiting the dosimetric comparison provides interesting insight to the treatment modality selection problem.

In our study, IMPT generally achieved similar dose sparing overall compared to the photon treatment methods, with the exception of the high doses to the femoral heads, due to proton entrance dose. Compared to the photon plans, IMPT is clearly better in PTV dose homogeneity and coverage. It also reduced maximal doses to the bladder, rectum, and anterior rectal wall. However, the advantage disappears when OAR volumes receiving high dose are considered. This is due to several factors. The most important one can be seen in Fig. 1, that the concave CTV is lateral to the anterior portion of the rectum, placing this volume along the proton beam direction and subject it to the increased distal penumbra dose. The second factor contributing to the rectum and bladder dose is the proton spot sizes ranging from 3.5 to 7.6 mm, creating less sharp beam edges in the directions perpendicular to the beams. Variable spot spacing may reduce the spot size related penumbra but has not been implemented in commercial planning systems.

The multivariate analysis shows that the magnitude of difference in dosimetric metrics of treatment modalities may depend on the OAR volume but not the relative relationships. For instance, the relative disadvantages and advantages of IMPT for V40 and V80, respectively, widen for larger rectum volumes. This information may be used to steer patient treatment if confirmed with a larger patient cohort. The bladder mean dose decreasing with increasing bladder volumes is intuitive. However, the similar decrease in the bladder maximum dose is less intuitive. It is possibly due to the distance between the bladder and the CTV also increasing with increasing bladder volume.

Between the two photon techniques, 4π plans are superior to VMAT plans with the exception of sigmoid colon dose and small differences in the maximum point doses to the bladder and rectum. The increase in dose to the sigmoid colon in the 4π plans is a result of non-coplanar beams delivering dose to superior and inferior structures. However, the off-plane dose is low and less of a concern in prostate treatment. This is consistent with previous studies comparing VMAT to 4π for a different prostate stereotactic body radiotherapy patient cohort [17]. Putting all three modalities together, one may make the observation that IMPT excels at reducing the maximal point dose to surrounding normal organs, reducing the low dose spillage and achieving a more homogeneous target dose. 4π improves the intermediate dose spillage compared to VMAT and achieves the lowest rectum volume receiving 40–70 Gy.

Different from the higher cost of proton, 4π delivery does not require new expensive equipment. Instead, it requires more sophisticated geometrical modeling to prevent gantry-patient collision. The delivery time of 4π plans involving a large number of beams can be excessively long in the manual mode. This limitation will be overcome using automating non-coplanar plan delivery [19].

## Conclusion

In comparison to coplanar VMAT and non-coplanar 4π plans for the prostate treatment, IMPT proton treatment plans showed benefits in integral dose, CTV coverage, homogeneity and maximum point OAR doses. IMPT is inferior in rectum and bladder volumes receiving intermediate to high doses, particularly to the 4π plans. The dosimetric benefits of 4π plans over VMAT are consistent with the previous publication. Specifically, increasing the organ weights of the rectum and bladder forces the plan to use more non-coplanar beams to move dose to the inferior and superior planes while similar increase in the coplanar VMAT plans was ineffective.

## Abbreviations

3D CPT: 3D conformal proton therapy
CAD: Computer assisted design
DVH: Dose volume histogram
IMPT: Intensity modulated proton therapy
IMRT: Intensity-modulated radiation therapy
OAR: Organ-at-risk
PBS: Pencil beam scanning
PTV: Planning target volume
SOBP: Spread-Out-Bragg-Peak
VMAT: Volumetric-modulated arc therapy
